# Influence of tramadol on bacterial burden in the standard neutropenic thigh infection model

**DOI:** 10.1038/s41598-022-24111-x

**Published:** 2022-11-15

**Authors:** K. Rox

**Affiliations:** 1 Department of Chemical Biology, Helmholtz Centre for Infection Research (HZI), Inhoffenstraße 7, 38124 Braunschweig, Germany; 2 German Center for Infection Research (DZIF), Partner Site Hannover-Braunschweig, 38124 Braunschweig, Germany

**Keywords:** Pharmacology, Pharmacodynamics, Drug discovery and development, Pharmacology

## Abstract

The neutropenic thigh infection model is one of the standard models in pharmacokinetic/ pharmacodynamic (PK/PD) characterization of novel antibacterials which are urgently needed due to the rise of antimicrobial resistance. The model enables to investigate PK/PD parameters crucial for translation of animal results towards humans. However, the neutropenic thigh infection model can result in moderate to severe discomfort of the animals, especially when high inocula are used. Tramadol has been proven to reduce pain effectively. This study investigates if tramadol influences the bacterial burden in the primary organ, the thighs, and organs affected by secondary seeding. Therefore, several strains of the ESKAPE pathogens, namely *S. aureus*, *P. aeruginosa*, *K. pneumoniae*, *E. coli*, *A. baumannii* and *E. faecalis* were examined. It was shown that tramadol did not influence the bacterial burden neither in thighs nor in organs affected by secondary seeding for the strains of *E. faecalis*, *S. aureus*, *P. aeruginosa*, *K. pneumoniae* and *E.coli* tested here, whereas secondary seeding seemed to be affected by tramadol for the tested strain of *A. baumannii*. Consequently, it was demonstrated that tramadol is an option to reduce discomfort in the untreated group for the strains of five out of the six tested ESKAPE pathogens and, thereby, contributes to the refinement of one of the standard PK/PD models.

## Introduction

Antimicrobial resistance is a silent and deadly pandemic with several million individuals affected every day around the world^1,2^. Therefore, antimicrobial resistance in the so-called ESKAPE pathogens, namely *Enterococcus faecium*, *Staphylococcus aureus*, *Klebsiella pneumoniae*, *Acinetobacter baumannii*, *Pseudomonas aeruginosa* as well as *Enterobacter* species, is of high concern. As a result, those pathogens have been listed as priority 1 (critical priority) or 2 (high priority) pathogens by the WHO^3,4^. New treatment options are urgently needed to enable to keep—or at least try to keep—pace with the pathogens developing new strategies to overcome the mechanisms of action of current antibiotics in the market^5^. During preclinical development, the evaluation of the performance of a novel antibiotic or even a completely new treatment option^6^, such as siderophores (e.g. cefiderocol^7^) or antimicrobial conjugates^8^, is key. For that purpose, standard pharmacodynamic models are used, such as the neutropenic lung and the neutropenic thigh infection model. Both of them are deployed to determine efficacy of novel treatment strategies in vivo and, thus, constitute an important milestone during early preclinical development^9^. Neutropenic mice are used as neutropenia hampers the immune response and prevents rapid clearance of the infection by the immune system^10^. Consequently, this allows to study and determine the PK/PD (pharmacokinetic/pharmacodynamics) index which helps to elucidate which pharmacokinetic parameter drives the effect of a novel drug, i.e. Cmax/MIC, time over MIC or AUC/MIC. The determination of the PK/PD index is crucial for effective translation into humans^11,12^.

The neutropenic thigh infection model, first described in the early 1950s^13^, results in moderate discomfort, especially for those animals just receiving vehicle or no treatment, due to inflammation and swelling of thighs in the course of the infection. This can lead to earlier sacrifice of animals when the humane endpoint is reached before the actual endpoint if very high inocula are used^14,15^. Of note, only one study deploying the neutropenic thigh infection model was published, in which a drug for pain relief, namely buprenorphine, was used^16^. However, no pain relieving drug has been investigated systematically in the neutropenic thigh infection model for its capacity to reduce pain as well as discomfort in the vehicle or untreated group *without* affecting bacterial burden in the primary organ as well as other organs possibly affected by secondary seeding, such as kidneys and lungs.

In this study, tramadol was chosen as a pain reducing agent. Tramadol belongs to the group of µ-receptor agonists, but has a different side effect profile than classical µ-receptor-agonists, such as morphine or fentanyl: it is a weak µ-opioid receptor agonist with non-opioid-related effects, e.g. on serotonin and noradrenaline transporters^17,18^. Moreover, it has been frequently used for animal experimentation for the treatment of pain in several indications^19^. Here, it is investigated if tramadol has an impact on bacterial burden in the primary organ, the thighs, but also if it has an impact on the bacterial distribution towards different organs compared to a group not receiving pain reducing treatment in the neutropenic thigh infection model. This assessment is important as it would enable to use tramadol in the vehicle or untreated group only, supposing that groups treated with antibiotic will not experience discomfort due to the active substance reducing bacterial burden. There are three reports about a potential antibacterial activity of tramadol against different ESKAPE pathogens without elucidating what the mechanism of action is^20–22^. Consequently, it is necessary to assess if tramadol exerts an effect at the dose and under the specific infection conditions in this study.

Therefore, several ESKAPE strains including Gram-positive as well as Gram-negative bacteria are tested, namely *S. aureus* (two different strains), *P.* *aeruginosa*, *K. pneumoniae*, *A. baumannii*, *E.coli* and *E. faecalis*. Moreover, it is assessed, specifically for *P. aeruginosa*, if there is a difference for the same strain when the model is extended up to 48 h. To the best of one’s knowledge, this is the first study investigating the use of a pain reducing agent only in the untreated control groups of the neutropenic thigh infection model with the aim to determine if the readout of the model was affected. Finally, it was shown that tramadol did not influence bacterial burden or secondary seeding to other organs for the strains of *E. faecalis*, *S. aureus*, *E. coli*, *P. aeruginosa* and *K. pneumoniae* used in this study. For the strain of *A. baumannii* used in this study no significant influence on bacterial burden in thigh was detected, but secondary seeding to other organs seemed to occur more frequently in the tramadol-treated group.

## Results

### Independent of the inoculum: tramadol treatment does neither affect bacterial burden in thighs nor organ distribution in the neutropenic thigh infection model with *E. faecalis* ATCC 29212

**Figure 1:**
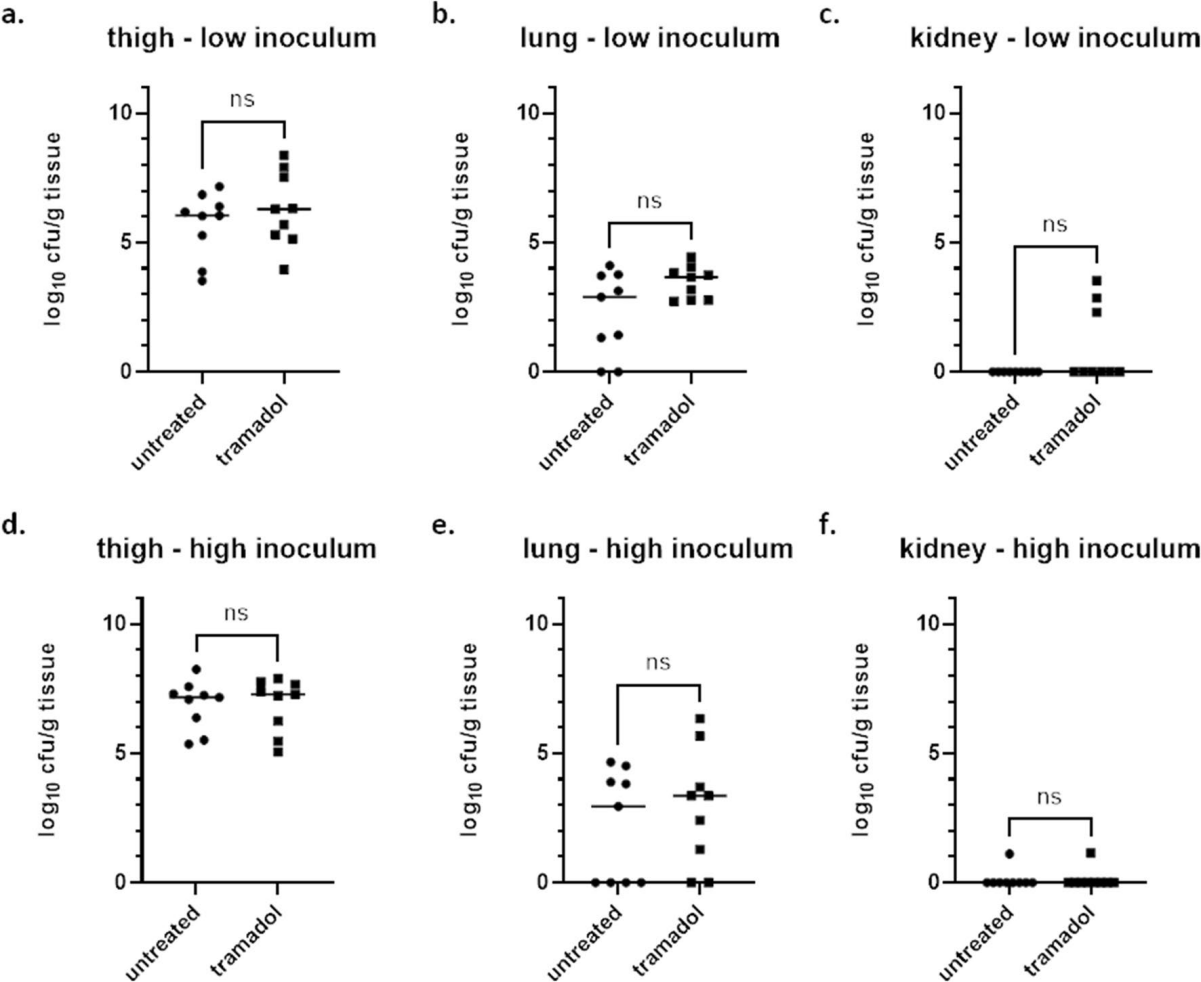
Bacterial burden in untreated and tramadol-treated groups in the neutropenic thigh infection model with two different inocula of *E. faecalis*. Bacterial burden expressed as cfu/g tissue was assessed for groups that were treated with either 20 mg/kg tramadol subcutaneously or left untreated (n = 9 per group). All groups were infected with *E. faecalis* at a low inoculum (1 × 10^7^ cfu/ml, **a**-**c**) or at a high inoculum (1 × 10^8^ cfu/ml, **d**-**f**). Bacterial burden was assessed in thigh (**a,d**), lung (**b,e**) and kidney (**c,f**). Statistical testing was performed using a Kolmogorov–Smirnov test. *Ns* not significant.

First, animals were infected after receiving cyclophosphamide at days -4 and -1 to render them neutropenic, with two different inocula of *E. faecalis* strain ATCC 29212, 1 × 10^7^ cfu/ml and 1 × 10^8^ cfu/ml. One group received tramadol at 20 mg/kg subcutaneously whereas the other group did not receive a pain reducing agent. Bacterial burden was assessed in thigh as the primary organ, but also determined in kidneys and lungs to evaluate if secondary seeding occurred. An inoculum of 1 × 10^7^ cfu/ml resulted in a median 6.0 log_10_ cfu/g tissue for the untreated and 6.3 log_10_ cfu/g tissue for the tramadol-treated group, whereas an inoculum of 1 × 10^8^ cfu/ml resulted in a median burden of 7.2 vs. 7.3 log_10_ cfu/g tissue for the untreated and the tramadol-treated group, respectively (Fig. 1a,d; Table 1, p = 0.7301 (low inoculum) and p = 0.9895 (high inoculum)). Consequently, tramadol treatment did not influence bacterial burden in the primary organ, the thighs. Moreover, it was determined if bacteria seed to secondary tissues in the course of the infection. Therefore, burden in lungs as well as in kidneys was assessed as well. For both inocula deployed in this study, only individual animals showed secondary seeding into the kidneys. No significant differences were detected between untreated and tramadol groups in kidneys (Fig. 1c,f). Additionally, bacterial loads in lung were determined. For the low inoculum of 1 × 10^7^ cfu/ml a median burden of 2.9 log_10_ cfu/g tissue was observed in the untreated group versus 3.7 log_10_ cfu/g tissue in the tramadol-treated group (Fig. 1b,e; Table 2). For the high inoculum of 1 × 10^8^ cfu/ml, only a median difference of about 0.4 log_10_ cfu/g tissue was shown in lung for the two groups. Neither for the low (p = 0.3517) nor for the high inoculum (p = 0.9737) this difference was significant. Moreover, bacterial loads in lung tissue were quite low (between 2 to 3 log_10_ cfu/g tissue). Finally, tramadol treatment did not affect the primary readout of the model, bacterial burden in the thighs, with the *E. faecalis* strain tested at different inocula sizes. No significant difference was detected between the untreated and the tramadol-treated group for organs affected by secondary seeding, either.

**Table 1:** Bacterial burden in thigh tissue.

Thigh	Untreated	Tramadol	Statistical comparison
*E. faecalis* (ATCC 29212, low inoculum)	6.05 ± 1.3	6.29 ± 1.5	0.7301
*E. faecalis* (ATCC 29212, high inoculum)	7.17 ± 1.3	7.29 ± 1.3	0.9895
*S. aureus* (MSSA, ATCC 29213)	8.26 ± 0.2	8.69 ± 0.3	0.1429
*S. aureus* (MRSA, ATCC 33591)	7.19 ± 0.9	7.77 ± 0.4	0.1429
*P. aeruginosa* (24 h, ATCC 27853)	5.02 ± 1.5	3.24 ± 0.6	0.1429
*P. aeruginosa* (48 h, ATCC 27853)	3.39 ± 1.5	3.52 ± 1.1	0.9119
*K. pneumoniae* (ATCC 43816)	5.31 ± 1.4	7.12 ± 1.5	0.4740
*A. baumannii* (ATCC 19606)	7.57 ± 0.6	7.60 ± 0.8	0.4740
*E. coli* (ATCC 25922)	9.25 ± 0.2	9.32 ± 1.2	0.9372

Median bacterial burden as well as standard deviation expressed as log_10_ cfu/g tissue in thigh for the untreated and the tramadol-treated group for *E. faecalis* ATCC 29212 (low and high inoculum), *S. aureus* strains ATCC 29213 and ATCC 33591, *P. aeruginosa* ATCC 27853 (readout after 24 and 48 h), *K. pneumoniae* ATCC 43816, *A. baumannii* ATCC 19606, *E. coli* ATCC 25922. Statistical testing was performed using a Kolmogorov–Smirnov test.

P values are indicated in the respective column.

**Table 2:** Bacterial burden in lung tissue.

Lung	Untreated	Tramadol	Statistical comparison
*E. faecalis* (ATCC 29212, low inoculum)	2.89 ± 1.7	3.66 ± 0.6	0.3517
*E. faecalis* (ATCC 29212, high inoculum)	2.95 ± 2.0	3.36 ± 2.3	0.9737
*S. aureus* (MSSA, ATCC 29213)	5.79 ± 1.0	5.95 ± 0.4	0.4740
*S. aureus* (MRSA, ATCC 33591)	5.29 ± 1.0	5.15 ± 0.8	0.9307
*P. aeruginosa* (24 h, ATCC 27853)	4.63 ± 2.6	4.87 ± 0.4	0.4740
*P. aeruginosa* (48 h, ATCC 27853)	4.69 ± 1.4	4.77 ± 0.7	0.9048
*K. pneumoniae* (ATCC 43816)	4.64 ± 1.8	4.37 ± 1.5	0.9370
*A. baumannii* (ATCC 19606)	0.0	3.56 ± 1.7	0.4156
*E. coli* (ATCC 25922)	5.90 ± 0.2	5.87 ± 0.9	0.9372

Median bacterial burden as well as standard deviation expressed as log_10_ cfu/g tissue in lung for the untreated and the tramadol-treated group for *E. faecalis* ATCC 29212 (low and high inoculum), *S. aureus* strains ATCC 29213 and ATCC 33591, *P. aeruginosa* ATCC 27853 (readout after 24 and 48 h), *K. pneumoniae* ATCC 43816, *A. baumannii* ATCC 19606, *E. coli* ATCC 25922. Statistical testing was performed using a Kolmogorov–Smirnov test.

P values are indicated in the respective column.

### Independent of the specific strain? Tramadol does not affect the readout for the neutropenic thigh infection model with *S. aureus* strains ATCC 33591 and ATCC 29213

**Figure 2:**
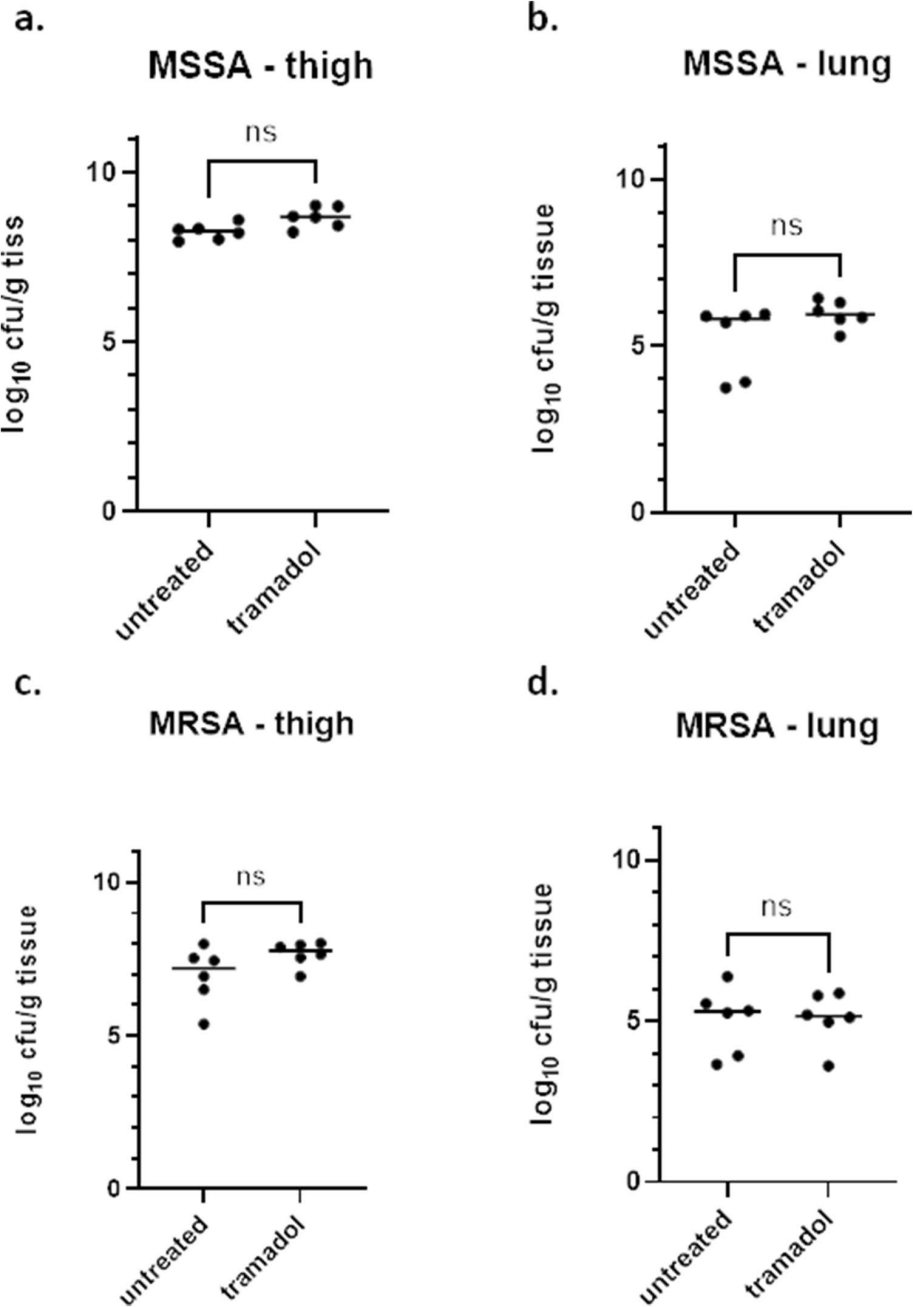
Bacterial burden in untreated and tramadol-treated groups in the neutropenic thigh infection model with two different strains of *S. aureus.* Bacterial burden expressed as cfu/g tissue was assessed for groups that were treated with either 20 mg/kg tramadol subcutaneously or left untreated (n = 6 per group). All groups were infected with MSSA (**a,b**) or MRSA (**c,d**). Bacterial burden was assessed in thigh (**a,c**) and lung (**b,d**). Statistical testing was performed using a Kolmogorov–Smirnov test. *Ns* not significant.

Next, the effect of tramadol in the neutropenic thigh infection model with *S. aureus* was assessed. Two different strains, a methicillin-sensitive *S. aureus* (MSSA, ATCC 29213) and a methicillin-resistant *S. aureus* (MRSA, ATCC 33591) strain, were used for this model, but with the same inoculum size. For the MSSA-strain a median burden of around 8.3 vs. 8.7 log_10_ cfu/g tissue was observed in thigh for the untreated and the tramadol-treated group, respectively. For the MRSA-strain a median burden of 7.2 vs. 7.8 log_10_ cfu/g tissue was detected for the untreated and the tramadol-group, respectively (Fig. 2a,c; Table 1). No significant difference in the bacterial burden in thighs for both strains was seen (p = 0.1429 (MRSA) and p = 0.1429 (MRSA)). With respect to secondary seeding, no bacteria were found in kidneys, whereas lung was affected in both strains with a burden of around 5.3 vs. 5.2 log_10_ cfu/g tissue for MRSA and around 5.8 vs. 5.9 log_10_ cfu/g tissue for MSSA for the untreated vs. the tramadol-treated group, respectively. Again, no difference was detected between tramadol- and untreated group for both strains (Fig. 2b,d; Table 2, p = 0.9307 (MSSA) and p = 0.4740 (MRSA))). Finally, for the two different *S. aureus* strains tested here, no impact of tramadol on bacterial burden in thighs or on distribution was observed.

### Independent of timing? Impact of tramadol in the neutropenic thigh infection model with *P. aeruginosa* at different readout time points

**Figure 3:**
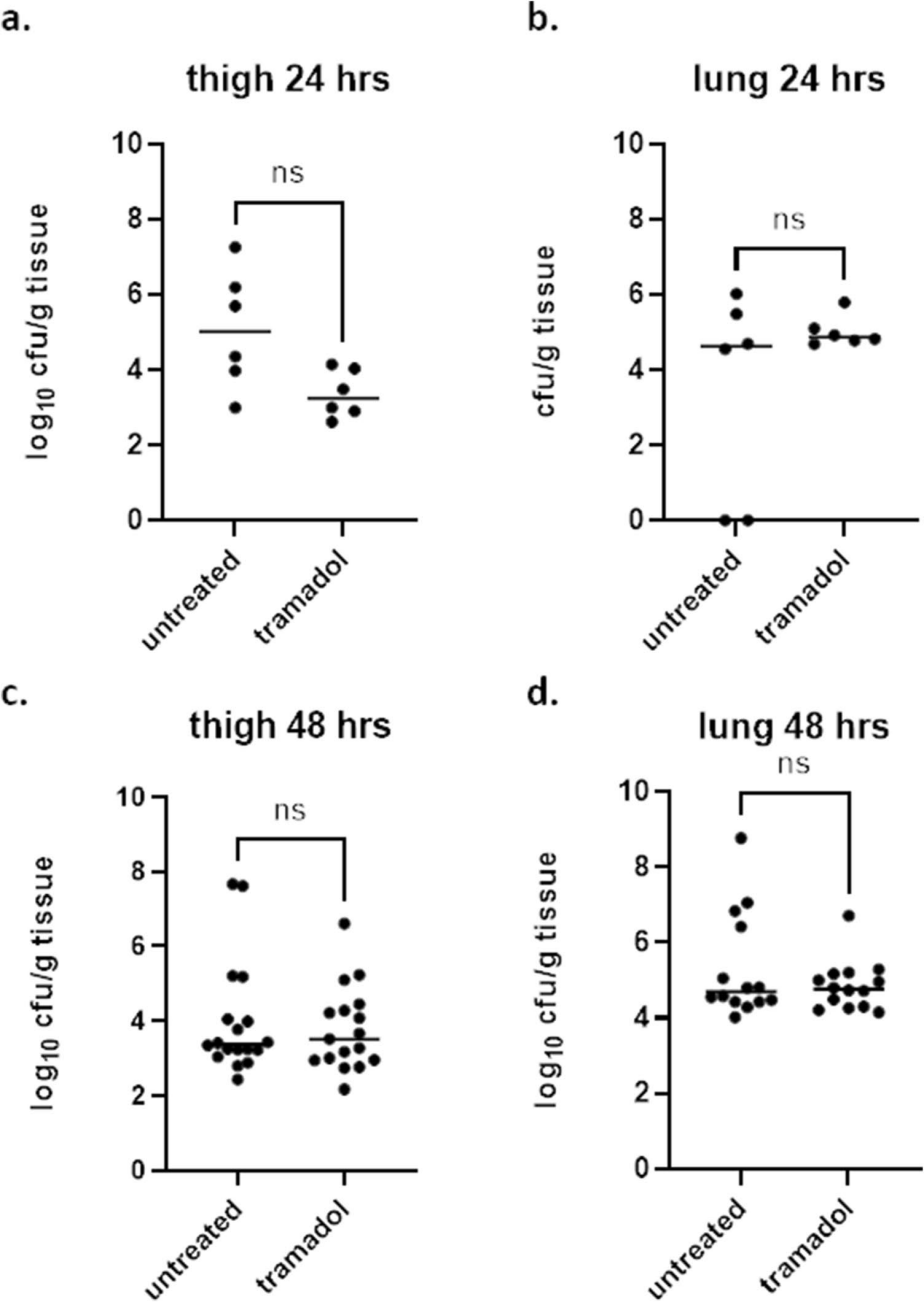
Bacterial burden in untreated and tramadol-treated groups in the neutropenic thigh infection model with two different readout time points of the same strain of *P. aeruginosa*. Bacterial burden expressed as cfu/g tissue was assessed for groups that were treated with either 20 mg/kg tramadol subcutaneously or left untreated. All groups were infected with *P. aeruginosa* either for 24 h ((**a,b**); n = 6 per group) or for 48 h ((**c,d**); n = 18 per group). Bacterial burden was assessed in thigh (**a,c**) and lung (**b,d**). Statistical testing was performed using a Kolmogorov–Smirnov test. *Ns* not significant.

Next, the impact of tramadol on the readout of the neutropenic thigh infection model was investigated when the same strain was used, but the animals were infected for either 24 h or 48 h. After 24 h a slightly higher burden of around 5.0 log_10_ cfu/g tissue in thigh tissue was detected in the untreated group compared to the tramadol-treated group (3.2 log_10_ cfu/g tissue) (Fig. 3a; Table 1). However, this was not significant upon statistical testing using a Kolmogorov–Smirnov-test which also takes into account the distribution in the individual groups (p = 0.1429). After 24 h a similar bacterial burden was found in lung tissue with of median 4.6 log_10_ cfu/g tissue (untreated) versus 4.9 log_10_ cfu/g tissue (tramadol) (Fig. 3b; Table 2, p = 0.4740). Next, the focus was on the possible impact of tramadol on the readout after 48 h of infection with the same strain. In thigh, a median burden of 3.4 log_10_ cfu/g tissue was seen in the untreated group compared to 3.5 log_10_ cfu/g tissue in the tramadol-treated group (Fig. 3c; Table 1, p = 0.9119). Moreover, in lung tissue a median burden of 4.7 log_10_ cfu/g tissue was found in the untreated treated group compared to 4.8 log_10_ cfu/g tissue in the tramadol-treated group (Fig. 3d; Table 2, p = 0.9048). Thus, no significant difference was detected in both tissues for both groups. In summary, tramadol treatment was similar to the untreated control groups when tested at different readout time points.

### Neutropenic thigh infection model with Gram-negative ESKAPE bacteria—impact of tramadol

**Figure 4:**
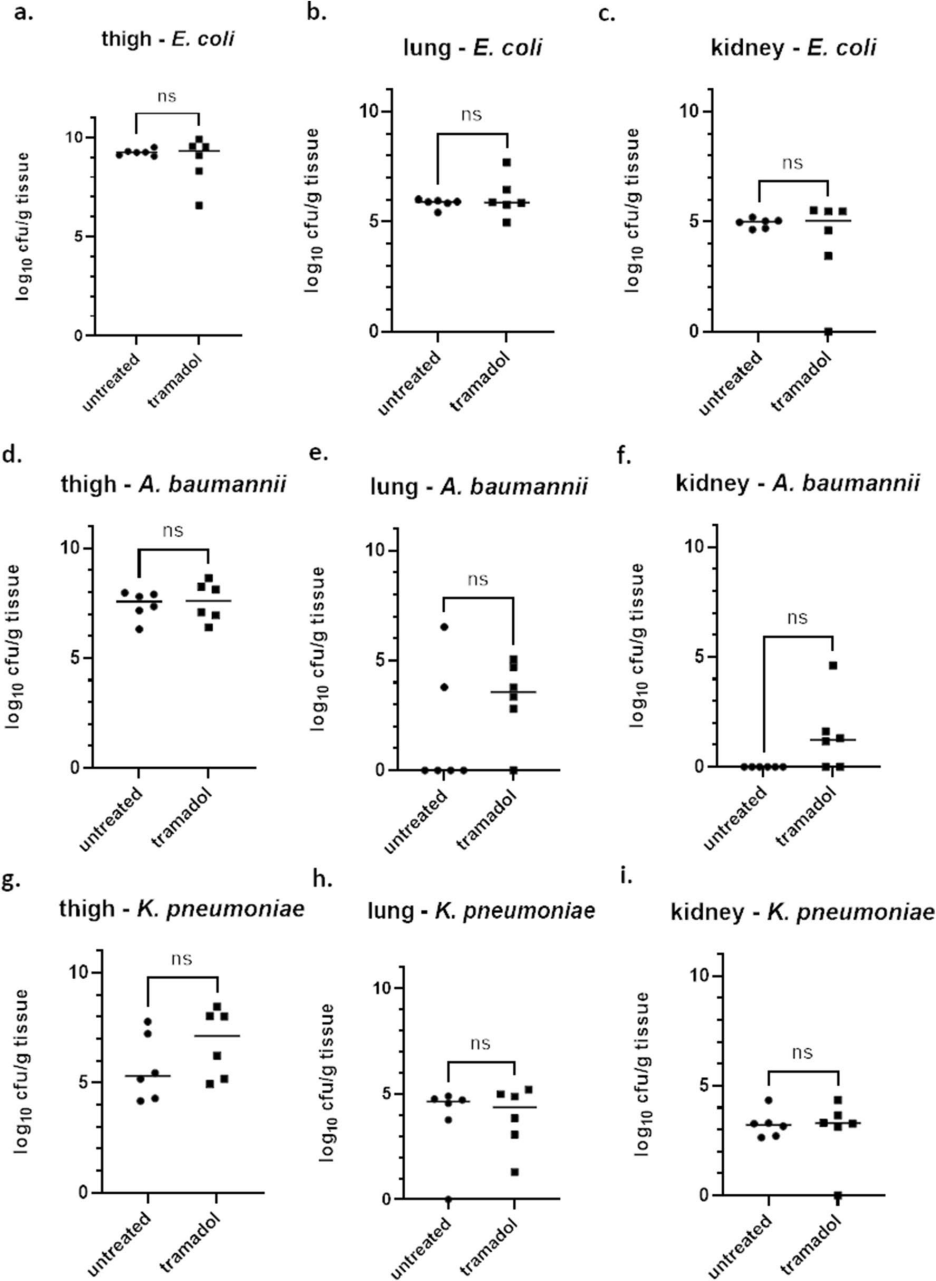
Bacterial burden in untreated and tramadol-treated groups in the neutropenic thigh infection model with three different species of the ESKAPE pathogens. Bacterial burden expressed as cfu/g tissue was assessed for groups that were treated with either 20 mg/kg tramadol subcutaneously or left untreated (n = 6 per group). All groups were either infected with *E. coli* (**a-c**) or with *A. baumannii* (**d-f**) or *K. pneumoniae* (**g-i**). Bacterial burden was assessed in thigh (**a,d,g**), lung (**b,e,h**) and kidney (**c,f,i**). Statistical testing was performed using a Kolmogorov–Smirnov test. *Ns* not significant.

In a next step, the influence of tramadol in the neutropenic thigh infection model with *E. coli*, *A. baumannii* and *K. pneumoniae* was determined. In the neutropenic thigh infection model with *E. coli* a median burden of around 9.3 log_10_ cfu/g tissue was observed in thigh for both groups (Fig. 4a; Table 1). Secondary seeding towards lung and kidneys occurred as well. In lung, a median burden of 5.9 log_10_ cfu/g tissue was found for both groups (Fig. 4b; Table 2), whereas in kidneys, a median burden of around 5.0 log_10_ cfu/g tissue was determined for both groups (Fig. 4c; Table 3). No significant differences were detected between both groups in the neutropenic thigh infection model with *E. coli*, neither in the primary readout organ, thigh (p = 0.9372), nor in the organs affected by secondary seeding (lung (p = 0.9372) and kidneys (p > 0.9999)).

**Table 3:** Bacterial burden in kidney tissue.

Kidney	Untreated	Tramadol	Statistical comparison
*K. pneumoniae* (ATCC 43816)	3.21 ± 0.6	3.30 ± 1.4	0.9307
*E. coli* (ATCC 25922)	5.00 ± 0.2	5.04 ± 2.1	> 0.9999

Median bacterial burden as well as standard deviation expressed as log_10_ cfu/g tissue in kidney for the untreated and the tramadol-treated group for *K. pneumoniae* ATCC 43816 and *E. coli* ATCC 25922. Statistical testing was performed using a Kolmogorov–Smirnov test. P values are indicated in the respective column.

Then, the effect of tramadol in the neutropenic thigh infection model with *A. baumannii* was investigated. Similarly to the *E. coli* model, no significant difference between the untreated and the tramadol group was observed with a median burden of around 7.6 log_10_ cfu/g tissue in thigh for both tested groups (Fig. 4d; Table 1, p = 0.4740). Secondary seeding to kidneys and lung tissue also took place to a lower extent. In lung, a median burden of 3.6 log_10_ cfu/g tissue was found in the tramadol group (Fig. 4e; Table 2), whereas in kidneys, a median burden of 1.2 log_10_ cfu/g tissue was detected in the tramadol group. However, not all animals were affected by secondary seeding into the kidneys or in the lungs. In the untreated group no secondary seeding into the kidneys was detected at all (Fig. 4f). Moreover, only two animals in the untreated group showed secondary seeding into the lung (Fig. 4e, p = 0.4156). No significant difference was detected in the different groups using the Kolmogorov–Smirnov-test.

Finally, the effect of tramadol in the neutropenic thigh infection model with *K. pneumoniae* was assessed. In thigh, a median burden of 5.3 log_10_ cfu/g tissue was determined in the untreated group versus 7.1 log_10_ cfu/g tissue in the tramadol group (Fig. 4g; Table 1, p = 0.4740). However, both groups exhibited a similar standard deviation. In lung, a median burden of 4.6 log_10_ cfu/g tissue was detected in the untreated group versus 4.3 log_10_ cfu/g tissue in the tramadol group (Fig. 4h; Table 2, p = 0.9307). In kidneys, the median burden for the untreated group was 3.2 log_10_ cfu/g tissue whereas the tramadol-treated group showed a median burden of 3.3 log_10_ cfu/g tissue (Fig. 4i; Table 3, p = 0.9307). The differences between the two tested groups were not significant in all tested organs. In conclusion, tramadol was similar to the untreated control group for the strains of the three bacterial species tested here with respect to bacterial burden in the primary, but also in organs affected by secondary seeding.

## Discussion

The neutropenic thigh infection model is a standard model for pharmacodynamic characterization of novel compounds and determination of the PK/PD driver via dose fractionation studies^9,11^. However, this model can cause moderate and, possibly at high inocula sizes, also severe discomfort for animals. This is indicated by studies showing that the vehicle-treated control group was euthanized before the actual planned endpoint due to animals of this group reaching the humane endpoint^14,15^. Therefore, in line with the 3R principle, established by Russell and Burch in the late 1950s, this study is the first one, to the best of one’s knowledge, that evaluated if a pain reducing agent can be used without influencing the bacterial burden in the primary readout organ, the thighs. One study was found that used buprenorphine three times a day for all groups in a neutropenic thigh infection model^16^. However, this study did not inform about the influence on bacterial burden in general. As all groups, also those investigated for their antibacterial potency, underwent buprenorphine, potential treatment effects on bacterial burden of buprenorphine per se might be negligible. Nevertheless, use of buprenorphine for all groups, including the treated ones, bears a high drug-drug-interaction potential, especially concerning drugs mainly metabolized via the CYP enzyme 3A4 as buprenorphine is mainly metabolized via this enzyme^23^. In this study, influence of tramadol on bacterial burden was investigated with the aim to enable treatment of only the untreated control group with tramadol. As a result, treatment groups do not need to receive tramadol-treatment for reduction of discomfort if substances show effect. This avoids drug-drug-interactions in study groups as a result of deployment of the pain reducing agent.

Dissemination in the neutropenic thigh infection model towards distant vital organs has been observed by others before^10,24^. The mechanism of this dissemination has not been determined. The thigh is a well perfused tissue so that it is conceivable that bacteria are capable to transition from the thigh to the blood stream and are then transported to other organs. As tramadol might influence the process of inflammation in the thigh, the perfusion of the non-inflamed thigh tissue could be different to the one in the untreated control group. One could speculate that this could result in a different degree of dissemination. Thus, it is crucial that tramadol does not influence the distribution of bacterial burden in the organs, potentially affected by secondary seeding, either.

In general, two classes of pain reducing agents appear to be suitable: cyclooxygenase (COX)-inhibitors and opioids. In the neutropenic thigh infection model described in this study, the kidneys were frequently affected by secondary seeding in the course of the infection. It is known that cyclooxygenase-inhibitors directly influence inflammation via the cyclooxygenase and, moreover, impact glomerular filtration via the cyclooxygenase-2^25–27^. It was shown in this study that secondary seeding from the primary infection site occurred for all species investigated here. For *E. faecalis* and *A. baumannii*, bacterial burden was detected in the kidneys of some individuals, whereas for *E. coli* and *K. pneumoniae*, a considerable burden was determined. Wang and colleagues showed that upon administration of a COX-inhibitor, endotoxins reduced the glomerular filtration rate, whereas this was not the case when endotoxins were administered without a COX-inhibitor^28^. This suggests that COX-inhibitors might contribute to increased severity and higher bacterial burden in the kidneys during the neutropenic thigh infection model. As a pain reducing agent is needed that does not influence the course of the infection, COX-inhibitors were excluded in this study right from the beginning. Therefore, tramadol was chosen as pain reducing agent: Due to different receptor affinities, tramadol does not bear the same potential to interfere with inflammation and pain compared to the full (morphine) or partial µ-opioid (buprenorphine) receptor agonists which also contributes to less side effects for tramadol^17,29,30^. Finally, tramadol has also been used extensively in animal models for pain so that doses administered via the subcutaneous route assure rapid analgesia lasting for several hours^31,32^ which was important for the neutropenic thigh infection model studied here.

In this study, only a small number of strains from different species was used to assess the potential effects of tramadol on bacterial burden. It was shown for several of the ESKAPE pathogens, Gram-positive as well as Gram-negative species, that tramadol did not influence the bacterial burden neither in thigh nor in organs affected by secondary seeding, such as lung and kidney. In general, all strains tested in this study caused secondary seeding at least into lung tissue. Only *A. baumannii* as well as both inocula of *E. faecalis* showed low inoculation of lung tissue with a bacterial burden lower than 4 log_10_ cfu/g tissue. With respect to kidney tissue, only infection with *K. pneumoniae* and *E. coli* resulted in a considerable burden.

By using different inocula sizes of the same strain of *E. faecalis*, it was demonstrated that the outcome of the infection upon treatment with tramadol was inocula-independent in both organs, thigh and lung. In this study, only two different inocula sizes were tested for *E. faecalis* which could be considered as a limitation. However, these inocula sizes are commonly used for neutropenic thigh infection models with *E. faecalis*^33^. Next, it was investigated if using two different strains of the same species, namely *S. aureus*, resulted in distinct outcomes. Again, no difference was observed for the primary organ thigh as well as for lung tissue affected by secondary seeding. This suggested that tramadol usage did not influence bacterial burden when different strains of the same species were used. It is evident that this has to be investigated further using several strains of the same species. Nevertheless, this first investigation is promising towards tramadol deployment for the untreated or vehicle group in the neutropenic thigh infection model.

When the thigh infection model has a prolonged endpoint at 48 h, as shown with *P. aeruginosa*, tramadol treatment does also not influence the bacterial burden. Nonetheless, tramadol-treatment resulted in a lower median burden in thigh tissue upon infection with *P. aeruginosa* for 24 h. This finding was not significant. Moreover, the untreated group showed a much higher standard deviation compared to the tramadol-treated group. Furthermore, no difference was detected between both groups in lungs after 24 h. There are two reports about the anti-pseudomonal and anti-staphylococcal activity of tramadol *in vitro*^20^ and in vivo^21^. In the in vivo study, tramadol was injected at high concentrations of about 62.5 mg/kg (based on an estimation of 20 g for the weight of Balb/c mice which were used in that study) directly into the site of infection^21^. Similarly, the in vitro study used concentrations of 12.5 mg/ml of tramadol to detect bacterial killing over 24 h^20^. By contrast, in the neutropenic thigh infection model, tramadol is injected at a different site and not injected together with the bacteria intramuscularly. A recent in vitro study also investigated the antibacterial properties of tramadol against the same strains of *E. coli*, *S. aureus* and *P. aeruginosa* used in this study and against a *K. pneumoniae* strain^22^. Only at concentrations of 500 µg/ml 100% killing of *K. pneumoniae* was achieved, whereas that concentration did not result in complete killing of *E. coli*, *S. aureus*, and *P. aeruginosa*^22^ suggesting that concentrations in the range of more than 500 µg/ml might be necessary to see complete eradication. Based on pharmacokinetic information about tramadol after subcutaneous administration^31^, these concentrations were not reached in this study. Therefore, a direct effect of tramadol on the bacterial infection in the neutropenic thigh infection model was unlikely.

In addition to the Gram-negative bacterium *P. aeruginosa*, also *E. coli*, *A. baumannii* and *K. pneumoniae* were tested. Whereas burden in all three organs (thigh, lung and kidney) was nearly identical for *E. coli* upon treatment with tramadol in comparison to untreated animals, *A. baumannii* showed the same burden only in thigh. For *A. baumannii* some individual animals were affected by secondary seeding. In lung tissue, it appeared that tramadol-treated animals seemed to bear a higher bacterial load compared to untreated animals. This was also reflected in kidney. It should be investigated further if tramadol treatment facilitates dissemination from the thigh tissue into other distant organs. Although the mechanism of secondary seeding is not elucidated, one could speculate that the reduced inflammation in thigh tissue as a result of effective tramadol treatment leads to a higher degree of secondary seeding. Although the differences in kidney and lung tissue were not statistically significant, one has to bear in mind that the analysis for detection of differences in bacterial load was primarily powered for thigh. Thus, it is possible that a potential effect of tramadol on the seeding in *A. baumannii* might not be detected as the secondary seeding to other organs goes in hand with a higher standard deviation. This would require a higher number of animals to retrieve statistically significant differences as well and could be subject to future studies. In the neutropenic thigh infection model with *K. pneumoniae* a higher bacterial load upon tramadol treatment was found in thighs which was not statistically significant due to the similar standard deviation in both groups. When looking into lung and kidney tissue, no differences were seen for both groups.

In conclusion, this study showed for a first small subset of different species and conditions, like different inocula sizes, different strains, different endpoints, that tramadol is a viable option to enhance animal welfare during the model and, thus, contributes to the refinement of one of the standard models in the field of PK/PD. Therefore, this study emboldens to use tramadol for those groups not receiving treatment to reduce discomfort as the bacterial burden is not affected. This study does not claim that tramadol does not exert an effect on bacterial burden for every specific bacterial strain and species used in neutropenic thigh infection models around the globe. However, it does encourage everyone using this model for determination of PK/PD effects of novel antibacterials to assess if tramadol is an option to reduce discomfort and to enhance the welfare of animals. Finally, this study fortifies that the usage of a pain reduction agent does not per se influence the outcome in this established in vivo model. It is rather necessary to investigate potential effects. This should encourage everyone using in vivo models resulting in pain, distress or discomfort for the animals to explore if a pain reduction agent is beneficial and able to reduce discomfort without influencing crucial parameters important for readout.

## Methods

### Strains

The following strains were used for in vivo studies: *S. aureus* ATCC 29213 (MSSA), *S. aureus* ATCC 33591 (MRSA), *K. pneumoniae* ATCC 43816, *P. aeruginosa* ATCC 27853, *E. coli* ATCC 25922, *A. baumannii* ATCC 19606, *E. faecalis* ATCC 29212.

### Preparation of the inoculum for infection with *S. aureus* strains ATCC 29213 (MSSA) and ATCC 33591 (MRSA)

The respective strain was streaked out from a glycerol culture onto a blood agar plate and incubated at 37 °C for 8 h. One colony was inoculated in 20 ml BHI medium (BD) and incubated for 15–16 h at 120 rpm and 37 °C. Then, the bacterial culture was diluted in fresh BHI medium to yield an OD600 of < 0.1 and incubated at 120 rpm at 37 °C until it reached an OD600 between 0.45 and 0.55. Next, the culture was centrifuged for 15 min at 4000 rpm and 4 °C. The pellet was washed twice using PBS and centrifuged for 15 min at 4000 rpm and 4 °C. Finally, the pellet was resuspended in PBS and the OD600 was adjusted to 1. Aliquots were prepared and frozen at − 80 °C. One day before infection one aliquot was plated onto BHI agar and incubated overnight at 37 °C. The following day, the colonies were counted to adjust the infection inoculum accordingly by dilution with PBS. For infection with *S. aureus* ATCC 29213 an inoculum of 1 × 10^6^ cfu/ml was used. For infection with *S. aureus* ATCC 33591 an inoculum of 1 × 10^6^ cfu/ml was used.

### Preparation of the inoculum for infection with *K. pneumoniae* ATCC 43816

The strain was streaked out from a glycerol culture onto a blood agar plate and incubated at 37 °C overnight. A few colonies were inoculated in a mixture of MHB diluted in water (one part MHB and nine parts water) and incubated for 14–15 h at 120 rpm and 37 °C. The following day, the culture was centrifuged at 4000 rpm and 4 °C for 15 min when it reached an OD600 between 0.5 and 0.7. Then it was washed twice with PBS and centrifuged for 15 min at 4000 rpm and 4 °C. Finally, the pellet was resuspended in PBS and the OD600 was adjusted to 1. Then, the inoculum was diluted 1:50,000 in PBS. For infection with *K. pneumoniae* an inoculum of 1 × 10^4^ cfu/ml was used.

### Preparation of the inoculum for infection with *E. coli* ATCC 25922

The strain was streaked out from a glycerol culture onto a blood agar plate and incubated at 37 °C overnight. A few colonies were inoculated in a mixture of TSB diluted in water (one part TSB and fourteen parts water) and incubated for 13–15 h at 130 rpm and 37 °C. The following day, the culture was centrifuged at 4000 rpm and 4 °C for 15 min when it reached an OD600 between 0.7 and 0.8. Then it was washed twice with PBS and centrifuged for 15 min at 4000 rpm and 4 °C. Finally, the pellet was resuspended in PBS and the OD600 was adjusted to 1. Then, the inoculum was diluted 1:100 in PBS. For infection with *E. coli* an inoculum of 3 × 10^6^ cfu/ml was used.

### Preparation of the inoculum for infection with *P. aeruginosa* ATCC 27853

The inoculum was prepared as follows: on day-1 the *P. aeruginosa* strain was streaked out onto a blood agar plate and incubated at 37 °C. Then one single colony was inoculated into MHB medium (diluted 1:2 in water) and incubated at 120 rpm and 37 °C. On day 0 bacteria were centrifuged for 15 min at 4000 rpm and washed twice in PBS. Then they were adjusted to an OD of 1 and diluted 1:5000 in PBS for the infection until 48 h and 1:1000 n PBS for the infection until 24 h. For infection with *P. aeruginosa* until 24 h an inoculum of 2 × 10^5^ cfu/ml was used whereas an inoculum of 3 × 10^4^ cfu/ml was used for an infection until 48 h.

### Preparation of the inoculum for infection with *A. baumannii* ATCC 19606

The strain was streaked out from a glycerol culture onto a blood agar plate and incubated at 37 °C overnight. A few colonies were inoculated in a mixture of MHB diluted in water (one part MHB and four parts water) and incubated for 14–15 h at 120 rpm and 37 °C. The following day, the culture was centrifuged at 4000 rpm and 4 °C for 15 min when it reached an OD600 between 0.7 and 0.8. Then it was washed twice with PBS and centrifuged for 10 min at 4000 rpm and 4 °C. Finally, the pellet was resuspended in PBS and the OD600 was adjusted to 1. Then, the inoculum was diluted 1:10 in PBS. For infection with *A. baumannii* an inoculum of 3 × 10^7^ cfu/ml was used.

### Preparation of the inoculum for infection with *E. faecalis* ATCC 29212

The strain was streaked out from a glycerol culture onto a blood agar plate and incubated at 37 °C for 8 h. One colony was inoculated in 20 ml BHI medium (BD) and incubated for 15–16 h at 120 rpm and 37 °C. Then, the bacterial culture was diluted in fresh BHI medium to yield an OD600 of < 0.1 and incubated at 120 rpm at 37 °C until it reached an OD600 between 0.3 and 0.6. Next, the culture was centrifuged for 15 min at 4000 rpm and 4 °C. The pellet was washed twice using PBS and centrifuged for 15 min at 4000 rpm and 4 °C. Finally, the pellet was resuspended in PBS and the OD600 was adjusted to 1. Aliquots were prepared and frozen at − 80 °C. One day before infection one aliquot was plated onto BHI agar and incubated overnight at 37 °C. The following day, the colonies were counted to adjust the infection inoculum accordingly by dilution with PBS. For infection with *E. faecalis* an inoculum of 1 × 10^8^ cfu/ml was used.

### Animals

The animal studies were conducted in accordance with the recommendations of the European Community (Directive 86/609/EEC, 24 November 1986). All animal procedures were performed in strict accordance with the German regulations of the Society for Laboratory Animal Science (GV- SOLAS) and the European Health Law of the Federation of Laboratory Animal Science Associations (FELASA). Animals were excluded from further analysis if sacrifice was necessary according to the humane endpoints established by the ethical board. All experiments were approved by the ethical board of the Niedersächsisches Landesamt für Verbraucherschutz und Lebensmittelsicherheit, Oldenburg, Germany. Animals were kept in individually ventilated cages with a 10 h/14 h dark/light cycle and had access to food and water ad libitum. The study is reported in accordance with the ARRIVE guidelines.

### Neutropenic thigh infection with *E. faecalis*, *S. aureus*, *P. aeruginosa*, *E. coli*, *K. pneumonia* or *A. baumannii* for 24 h

Male, 6 weeks-old, CD-1 mice (Charles River, Germany) were rendered neutropenic by administration of 150 mg/kg and 100 mg/kg cyclophosphamide intraperitoneally on day -4 and -1, respectively. Mice were grouped as follows per strain (n = 6 for *S. aureus*, *P. aeruginosa*, *E. coli*, *K. pneumonia* or *A. baumannii* and n = 9 for *E. faecalis*): (1) untreated group, (2) tramadol group (receiving 20 mg/kg subcutaneously t = 0 h post infection). On the day of infection (day 0), mice received 30 µl of the respective strain into each lateral thigh under isoflurane anesthesia. 24 h after infection, mice were euthanized, blood was removed from the heart, lung and thighs were aseptically removed. 24 h after infection the clinical score of every individual animal was assessed. Clinical scoring comprised assessment of different parameters such as spontaneous behavior, posture, appearance, provoked behavior and body weight. Each parameter was assessed with a score of 0 to 3 (ascending severity from 0 to 3). The humane endpoint was reached when the clinical score of one parameter was 3 or if the clinical score was higher than 8. Whole blood was collected into Eppendorf tubes coated with 0.5 M EDTA and immediately spun down at 13.000 rpm for 10 min at 4 °C. The plasma was transferred into a new Eppendorf tube and then stored at − 80 °C until analysis. Organs were homogenized in 0.9% NaCl-solution and plated onto agar plates in duplicates in serial dilutions and incubated at 37 °C for 24 h.

### Neutropenic thigh infection model with *P. aeruginosa* for 48 h

Male, 6 weeks-old, CD-1 mice (Charles River, Germany) were rendered neutropenic by administration of 150 mg/kg and 100 mg/kg cyclophosphamide intraperitoneally on day -4, -1 and + 1, respectively. The *P. aeruginosa* strain ATCC 27853 was used. The inoculum was plated onto MHB agar plates in serial dilutions and incubated at 37 °C. Mice were grouped into the following (n = 18 for each group): (1) untreated group, (2) tramadol group (receiving 20 mg/kg tramadol subcutaneously t = 0 and 24 h post infection). On the day of infection (day 0), mice received 30 µl of the *P. aeruginosa* strain ATCC 27853 into each lateral thigh (inoculum: 5 × 10^4^ cfu/ml) under isoflurane anesthesia. 48 h after infection, mice were euthanized, blood was removed from the heart, lung and thighs were aseptically removed. 24 and 48 h after infection the clinical score of every individual animal was assessed. Clinical scoring comprised assessment of different parameters such as spontaneous behavior, posture, appearance, provoked behavior and body weight. Each parameter was assessed with a score of 0 to 3 (ascending severity from 0 to 3). The humane endpoint was reached when the clinical score of one parameter was 3 or if the clinical score was higher than 8. Whole blood was collected into Eppendorf tubes coated with 0.5 M EDTA and immediately spun down at 13.000 rpm for 10 min at 4 °C. The plasma was transferred into a new Eppendorf tube and then stored at − 80 °C until analysis. Organs were homogenized in 0.9% NaCl-solution and plated onto MHB agar plates in duplicates in serial dilutions and incubated at 37 °C for 24 h.

### Statistical testing

Testing for statistical significance was performed using a Kolmogorov–Smirnov-test (unpaired, two-tailed) using GraphPad Prism 9.3.1 software. Results were considered statistically significant when p-values were below 0.05 (95% confidence interval).

## Data Availability

The author confirms that the data supporting the findings of this study are available within the article.
